# Development of a Multimorbidity Illness Perceptions Scale (MULTIPleS)

**DOI:** 10.1371/journal.pone.0081852

**Published:** 2013-12-20

**Authors:** Chris J. Gibbons, Cassandra Kenning, Peter A. Coventry, Penny Bee, Christine Bundy, Louise Fisher, Peter Bower

**Affiliations:** 1 NIHR Collaboration for Leadership in Applied Health Research and Care for Greater Manchester, University of Manchester, Manchester, United Kingdom; 2 NIHR School for Primary Care Research, NIHR Greater Manchester Primary Care Patient Safety Translational Research Centre, Manchester Academic Health Science Centre (MAHSC), University of Manchester, Manchester, United Kingdom; 3 School of Nursing, Midwifery and Social Work, University of Manchester, Manchester, United Kingdom; 4 Manchester Centre for Health Psychology, Institute of Inflammation and Repair, University of Manchester, Manchester, United Kingdom; McGill University, Canada

## Abstract

**Background:**

Illness perceptions are beliefs about the cause, nature and management of illness, which enable patients to make sense of their conditions. These perceptions can predict adjustment and quality of life in patients with single conditions. However, multimorbidity (i.e. patients with multiple long-term conditions) is increasingly prevalent and a key challenge for future health care delivery. The objective of this research was to develop a valid and reliable measure of illness perceptions for multimorbid patients.

**Methods:**

Candidate items were derived from previous qualitative research with multimorbid patients. Questionnaires were posted to 1500 patients with two or more exemplar long-term conditions (depression, diabetes, osteoarthritis, coronary heart disease and chronic obstructive pulmonary disease). Data were analysed using factor analysis and Rasch analysis. Rasch analysis is a modern psychometric technique for deriving unidimensional and intervally-scaled questionnaires.

**Results:**

Questionnaires from 490 eligible patients (32.6% response) were returned. Exploratory factor analysis revealed five potential subscales ‘Emotional representations’, ‘Treatment burden’, ‘Prioritising conditions’, ‘Causal links’ and ‘Activity limitations’. Rasch analysis led to further item reduction and the generation of a summary scale comprising of items from all scales. All scales were unidimensional and free from differential item functioning or local independence of items. All scales were reliable, but for each subscale there were a number of patients who scored at the floor of the scale.

**Conclusions:**

The MULTIPleS measure consists of five individual subscales and a 22-item summary scale that measures the perceived impact of multimorbidity. All scales showed good fit to the Rasch model and preliminary evidence of reliability and validity. A number of patients scored at floor of each subscale, which may reflect variation in the perception of multimorbidity. The MULTIPleS measure will facilitate research into the impact of illness perceptions on adjustment, clinical outcomes, quality of life, and costs in patients with multimorbidity.

## Introduction

Individuals with multiple co-existing long-term conditions (so-called ‘multimorbidity’) are increasingly common [1]–[5]. The prevalence of multimorbidity increases with age [6] and socio-economic deprivation [7]. Management of multimorbidity will be a critical challenge facing health care services worldwide [8], [9].

The psychological processes underlying patient adjustment to multimorbidity are of key interest to health care researchers, not least because of their potential to predict clinical, quality of life and cost outcomes [10]. One psychological process of particular interest is the development of *illness perceptions* (also known as *illness representations*) [11], [12]. Illness perceptions are beliefs about the cause, nature and management of illness, which enable patients to make sense of their conditions [13] and better cope with the associated challenges. The Common Sense Model of illness perceptions hypothesises that patients form both cognitive and emotional perceptions of illness [11]. Cognitive components include illness *identity* (illness label and symptoms); *cause* (e.g. whether a disorder has a genetic pre-disposition or is caused by lifestyle); *timeline* (the duration of the condition and whether it is acute or chronic); perceptions of *cure or control* (potential for cure or ability to manage symptoms) and *consequences* (beliefs about outcomes). Emotional perceptions concern emotional responses to both the illness (such as anxiety, depression and anger) and to its outcomes (*e.g.* fear for future complications) [14].

Measuring and understanding illness perceptions is important as they are capable of predicting health behaviours [15]–[17] and there is emerging evidence that interventions designed to *modify* illness perceptions can improve health outcomes for patients with single conditions [18], [19].

There is a paucity of research evaluating illness perceptions in patients with multimorbidity. Qualitative investigation suggests that illness perceptions formed by multimorbid patients may differ from those held by patients with a single long-term condition in two main ways. Firstly, multimorbidity may change patient's perceptions of individual conditions (*i.e.* their perceptions of the identity, cause and timeline of a single condition may change when they have a co-existing condition). Secondly, patients with multimorbidity may demonstrate *additional* perceptions concerning multimorbidity itself, which may include perceptions of treatment burden from multimorbidity, causal relationships between conditions, priorities among conditions as well as synergies and antagonisms between conditions [20]. However, research that seeks to assess the clinical utility of illness perceptions in multimorbidity is currently limited by the lack of an appropriate measurement tool. The objective of this study is to investigate the psychometric and scaling properties of a novel measure of illness perceptions in multimorbidity using Rasch analysis [21].

## Methods

### Measure design

A conceptual model was developed using published theory on illness perceptions and the common sense model, existing illness perception scales such as the Illness Perception Questionnaire (IPQ and IPQ-R) [17], a meta-synthesis of patient experience of the psychological impacts of diabetes and depression [22], and exploratory qualitative work assessing illness perceptions in the presence of multimorbidity [20]. From these resources 53 candidate items for a measurement scale were developed. Face validity was examined with a sample of 11 patients with multimorbidity using cognitive interviewing [23]. Candidate items were screened for jargon, value-laden words, overlapping questions, excessive length and ambiguity [24], leading to the removal of 11 items. The 42 remaining items that comprised the nascent scale were conceptually grouped into five dimensions: *interactions* between conditions; *priorities* (relative importance of conditions); *coherence* (impact of multimorbidity on understanding of conditions); *synergies* (how management of one condition impacts on others); and *consequences* (impact of multimorbidity). All items were scored on a 6-point Likert scale from 0 ‘strongly disagree’ to 5 ‘strongly agree’.

### Questionnaire administration

We identified 1500 patients from four general practices in Greater Manchester that had two or more of five exemplar conditions (diabetes, depression, osteoarthritis, chronic obstructive pulmonary disease and coronary heart disease). Patients were identified from Quality and Outcomes Framework (QOF) [25] registers at participating family care centres. These registers offer a standardised format for the classifying diseases and form part of a pay-for-performance scheme for general practitioners in England [26]. These conditions were chosen because they are prevalent and vary in their symptom profile and treatments, thus maximising variation in potential illness burden and perceptions of multimorbidity. Patients were still included if they had other long-term conditions additional to the exemplar conditions. Patients with terminal illness or severe and enduring mental health problems were excluded. Recruitment was conducted from June 2012 until August 2013.

A randomly-selected sample of 40% baseline-completers was asked to complete the new measure again, one month after baseline, to assess test-retest reliability.

#### Other measures

In order to assess construct validity, participants completed the Brief Illness Perceptions Questionnaire (bIPQ) [14], The Hospital Anxiety and Depression Scale [27] and the Health Education Impact Questionnaire (heiQ) [28]. Because the bIPQ was designed for use with single conditions and not multimorbidity patients were asked to nominate the condition they felt was the most disabling, and complete the bIPQ in relation to that condition.

Deprivation level was calculated using the participant's home postcode, which was then entered onto into the English Indices of Deprivation Database to assign deprivation level [29]. Additional comorbidity information was collected using the Bayliss self-report measure. The measure provides both an indication of the number of chronic conditions and subjective disease burden associated with those conditions. It is correlated strongly with other measures of comorbidity; including the Charlson Comorbidity Index and the RxRisk score [30].

### Analysis Procedure

Scale development was carried out using factor analysis for initial exploration of dimensionality [31] and Rasch analysis [21] to further evaluate the psychometric and scaling properties of the MULTIPle scales.

### Factor Analysis

Factor analysis is employed to establish initial dimensionality, prior to more rigorous tests of dimensionality within Rasch analysis [31]. The number of factors to be retained was calculated by comparing experimental eigenvalues against eigenvalues created at random using a Monte Carlo Analysis Protocol [32]. If the experimental eigenvalue for a factor was greater than eigenvalue created at random, then the factor was retained. Given the likelihood that factors were correlated, exploratory factor analysis was used with an oblique rotation [33]. Factor analysis gives an initial indication of unidimensionality prior to more rigorous tests of dimensionality during Rasch analysis, as such items were retained if they exhibited a factor loading ≥0.30. Items that cross-loaded onto more than one factor were removed [34].

### Rasch Analysis

Following initial exploration of dimensionality using Factor Analysis [31], scale data were analysed using Rasch analysis [21]. Rasch analysis is a modern psychometric method used to develop and validate questionnaires that satisfy the demands of fundamental measurement, and are therefore capable of creating interval-level measurement [21], [35]. Interval-level measurement is a necessity if accurate comparisons are to be made between patients or across patients over time, or if mathematical operations are to be carried out with questionnaire data [35]. The Rasch model was originally developed for use in educational testing, and proposes that the probability of a person correctly responding to a given question on a test is a logistic function between the ability of that person and the difficulty of the question they are responding to. In the context of multimorbidity illness perceptions, ‘ability’ and ‘difficulty’ might relate to ‘impact of multimorbidity’ experienced by the patient and ‘impact of multimorbidity’ expressed by the item. Rasch analysis provides additional tests alongside traditional assessments of validity and reliability, including local independence of items, differential item functioning, item category threshold order, unidimensionality and scale targeting [31], [34], [36]. These additional psychometric criteria make the Rasch model an attractive tool for questionnaire development [35] and are briefly described below. A more comprehensive review of the Rasch model is available elsewhere [34].

For analyses in the current study the unrestricted ‘partial credit’ Rasch polytomous model was used with conditional pair-wise parameter estimation [37] as response categories were polytomous (*i.e.* they had more than two response options). Analyses were conducted using SPSS 18 [38] and RUMM2030 [39].

#### Fit to the Rasch model

Data are required to meet Rasch model expectations, and a number of indicators are used for this purpose. Overall scale fit to the Rasch model is indicated by a non-significant summary chi-square statistic. In addition to assessing scale fit to the Rasch model, person and item fit to the Rasch model can be evaluated using residual mean values, where the summary fit standard deviation should be within ±1.4, with individual person and item residuals in the range of ±2.5. The closer these indicators are to zero, the better the fit the Rasch model [34].

#### Local dependency

An assumption of the Rasch model is that items are locally independent, conditional upon the phenomenon being measured. For example, two items that ask “I can walk unassisted for 10 meters” and “I can walk 100 m without help” are locally dependant, as affirmation of the second statement necessarily also affirms the first. Local dependency is identified by significant residual item correlations.

#### Differential Item Functioning (DIF) [36]

Differential Item Functioning (DIF) occurs when different demographic groups within the sample (*e.g*. males and females) respond in a different way to a certain question, given the same level of the underlying phenomenon. Two types of DIF can be identified; uniform and non-uniform. Uniform DIF occurs where there is a systematic difference across groups; for example respondents over 70 years old scoring lower than respondents under 70 on an item, irrespective of the level of the phenomenon (*e.g.* perceived of burden of multimorbidity) being measured by the scale. Non-uniform DIF occurs where groups respond differently to an item at certain levels of the attribute being measured; for example men scoring higher than women on an item when they have low levels of an phenomenon being measured, but lower when they have a high level of that phenomenon. Analysis of variance (ANOVA, 5% alpha with Bonferroni correction) is used to assess both uniform and non-uniform DIF.

#### Item Category Thresholds

The Rasch model also allows for a detailed analysis of the way in which respondents understand response categories. For example, in the case of a Likert-style response, respondents may have difficulty differentiating between ‘Agree’ and ‘Agree Somewhat’. In instances where there is too little discrimination between two response categories on an item, collapsing the categories into one response option can often improve scale fit.

#### Reliability

We assessed reliability in three ways. The extent to which items distinguish between distinct levels of the phenomenon being measured (*e.g.* ‘interactions between conditions’) was measured using Person Separation Index and Cronbach's alpha, which both range from 0 to 1. The value of 1 indicates perfect reproducibility of person estimates. For PSI, values of .7 are usually considered a minimal value for group use and .85 for individual patient use. Person Separation Index and Cronbach's alpha are analogous, however as the PSI is calculated using a non-linear transformation of raw scores, the effect of this is that the error variance will increase for PSI values where scores are taken from close to the extremes of the scale. In contrast, alpha values remain stable as scale scores approach extremes.

A randomly-selected sample of 40% of the baseline-completers was asked to complete the new measure a second time, one month after baseline to assess test-retest reliability.

#### Unidimensionality

Unidimensionality is present when each of the items within the scale measure the same phenomenon (*e.g.* ‘interactions between conditions’). To assess unidimensionality, two estimates are derived from items forming high positive and high negative loadings on the first principal component of the residuals. These are compared using t-tests. The number of significant t-tests outside the ±1.96 range indicates whether the scale is unidimensional or not. Generally, if less than 5% of t-tests are significant, the scale is considered to be unidimensional (or the lower bound of the 95% binomial confidence interval is below 5%) [37].

#### Scale Targeting

Scale targeting refers to the degree to which a measure is calibrated to the population that completes it, and is assessed by comparing person and item locations. A graph is produced in which the level of trait displayed by respondents (person location) is plotted in logits above the *x*-axis of the graph and the level of the trait that the items measure (item location); also measured in logits, is displayed below the *x*-axis. Graphing person and item locations in this way allows comparison of the scale's ability to measure the level of the phenomenon (*e.g.* ‘treatment burden from multimorbidity’), and facilitate comparison between the ‘amount’ of phenomenon that is displayed by the respondents and the range of that phenomenon adequately measured by the scale.

#### Higher-order scales

The presence of a higher-order scale may be investigated where distinct unidimensional scales can be summated to form a higher-order unidimensional measure. For example, previous studies [34], [40] have used this technique to confirm the presence of a higher-order factor of ‘Psychological distress’ for the two-factor Hospital Anxiety and Depression Scale (HADS) [27]. It is common practice to group items from each unidimensional factor into one ‘super-item’ or testlet, to avoid any analytical issues with local dependency when evaluating the presence of a higher order scale [41], [42].

In the event that more than one of the MULTIPleS subscales satisfy the demands of the Rasch model, the presence of a unidimensional higher-order summary scale was evaluated. Evaluation of a higher-order scale was conducted by adding items from each subscale into a single ‘testlet’ and then evaluating fit to the Rasch model for all of the items together, with items from individual subscales collapsed together into testlets.

### Test-retest reliability

Test-retest reliability was assessed by comparing scores obtained as baseline and at the one month follow-up. Previous researchers have found that illness perceptions appear to change somewhat over time, and have found acceptable test-retest reliability to be in values greater than 0.50, which was our criterion in the current study [17].

### External construct validity

External construct validity will be assessed by comparison between the nascent scale and comparator measures of single condition illness perceptions (The Brief Illness Perceptions Questionnaire – bIPQ) [14], health education (The Health Education Impact Questionnaire - heiQ) [28] and psychological distress (The Hospital Anxiety and Depression Scale - HADS) [27]. As no measure of illness perceptions related to multimorbidity currently exists, external construct validity must be assessed using questionnaires that measure theoretically related concepts. The three measures chosen are expected to demonstrate empirical associations with illness representations in multimorbidity as captured in the final version of the nascent questionnaire.

Comparisons between measures will be made using Pearson's correlation coefficient or Spearman's Rho depending on distribution of scale scores. The magnitude of relationships between the nascent scales and the comparator measures are expected to vary on the similarity of measured constructs. Correlation values starting at .50 will be considered indicative of construct validity where constructs are theoretically similar.

### Sample size

For Rasch analysis, a sample size of 243 allows for accurate person and item estimates, irrespective of scale targeting [43]. If enough data is collected (*circa.* 500 cases) then two samples may be independently analysed using Rasch analysis as evaluation and a validation samples. Assessing congruence between an evaluation and validation Rasch analysis will increase confidence that any data that is fit to the Rasch model is not simply an artefact of the dataset.

### Missing data

Where questionnaires are returned with greater than 40% data missing they will be excluded from all analyses. No missing data were imputed prior to Factor and Rasch analyses of individual items. For missing data in external construct validity analysis using total scale scores, mean imputation was used.

### Ethical Support

Ethical approval for the current study was granted by Greater Manchester North Ethics Committee on 12/09/2011 (ref: 11/NW/0563).

## Results

In total 36% (n = 539) of patients responded to the postal questionnaire. Of the 539 responders, 40 were excluded as they did not recognise that they had 2 of the long term conditions that made up our definition of multimorbidity and 9 because the questionnaire was not sufficiently completed (>40% missing data). Table 1 displays demographic statistics for participants in the study. Data from the main sample was randomly split into ‘evaluation’ and ‘validation’ samples (n = 254 and 236). A total of 173 patients (88%) returned the retest questionnaire.

**Table 1:** Demographic and comorbidity information for study participants (n = 490).

	% or M ± SD
Age (years)	70±10
Female	51%
Employed	13%
Retired	69%
Index of Multiple Deprivation	25±18
Number of exemplar conditions	2.3±0.8
Number of total conditions*	7.3±3.2
Patients with 2–5 comorbidities*	34.20%
Patients with 6–10 comorbidities*	50.20%
Patients with 11+ comorbidities*	15.60%
Disease burden score*	23.5±12.5
Chronic Obstructive Pulmonary Disease	35%
Coronary Heart Disease	50%
Depression	41%
Diabetes Mellitus	45%
Osteoarthritis	52%

* = Information taken from Bayliss comorbidity measure.

### Factor Analysis

Five factors had eigenvalues greater than the eigenvalue created at random using the Monte Carlo protocol and were retained.

The rotated five-factor solution is presented in Table 2 (short item versions are given in the table).

**Table 2:** Factor loadings for MULTIPleS items.

	1	2	3	4	5
Makes me sad	0.91				
Makes me angry	0.87				
Makes me more unhappy	0.87				
Makes me angry	0.86				
Makes me more irritable	0.74				
Overwhelmed with coping	0.73				
Makes managing a struggle	0.68				
Makes it harder to cope	0.65				
Hard to manage other conditions		0.84			
Difficult to get best treatment		0.65			
Don't like mixing medications		0.64			
Difficult to take all medicines		0.63			
Makes treatment less effective		0.63			
I take advice for some conditions more than others		0.59			
Medication has caused me problems		0.51			
Makes control difficult			0.82		
More worrying than others			0.73		
Dominates the others			0.65		
More impact on my life			0.5		
More serious than others			0.38		
Deal with one at a time			0.35		
One condition has caused another				0.89	
The causes are linked				0.86	
Conditions interact with each others				0.36	
Managing conditions reduced my social life					0.79
Difficult to carry out usual activities					0.63
Time managing has limited my activities					0.59
Eigenvalue	16.34	2.386	1.191	1.7	1.51

Factor Eigenvalues: 1 = 16.34, 2 = 2.86, 3 = 1.19, 4 = 1.70, 5 = 1.51.

Data were severely skewed (>±1.0) for all of the constituent items of the MULTIPle scales and as such, factor analysis was conducted with Principal Axis Factoring (PAF) extraction, with oblique promax rotation. Principal axis factoring is the recommended extraction for data that are severely skewed [33].

Factors were labelled as follows: 1- *Emotional representations*, 2- *Treatment burden*, 3- *Prioritising conditions*, 4- *Causal links*, and 5-*Activity limitations*.

### Rasch Analysis

#### Emotional representations scale

Initial model fit for the ‘Emotional representations’ scale was poor (χ^2^(24) = 44.98, p<0.0001). Misfit appeared to be driven by the high fit residual for item 2 ‘Difficult to cope’. Removal of item 2 led to improved fit characteristics for the scale, however item 19 “Overwhelmed” displayed uniform DIF by age group, meaning that older patients were more likely to agree with the item than younger patients (*p*<0.0001). Removal of item 19 resulted in excellent model fit including good local dependency, absence of DIF, correctly ordered thresholds and was reliable and unidimensional (Table 3, Analysis 1).

**Table 3:** Summary fit statistics for MULTIPleS analyses in evaluation and validation samples.

Analysis	Scale	# of items	n	Item Residual		Person Residual		Chi Square		Reliability		Unidimensional T-test %	% Extreme
				Mean	±SD	Mean	±SD	Value	*p*	PSI	α	t-test (%)	scores
	Original Sample												
1	Emotional	6	254	0.46	0.72	−0.48	1.3	29.03	0.05	0.81	0.93	0.79%	21.00%
2	Treatment burden	6	254	0.26	1.52	−0.52	1.53	22.79	0.2	0.7	0.9	0.79%	32.00%
3	Prioritization	4	254	0.26	0.7	−0.4	1.08	21.63	0.04	0.64	0.79	0.39%	16.00%
4	Causal links	3	254	0.76	0.56	−0.54	1.3	8.51	0.48	0.49	0.79	0.00%	33.00%
5	Activity limitation	3	254	0.4	0.19	−0.85	1.75	8.9	0.44	0.65	0.8	0.00%	29.00%
6	Summary scale	22	254	−0.16	1.55	−0.34	0.96	24.89	0.22	0.81	0.85	2.78%	1.00%
	Validation Sample												
7	Emotional	6	235	0.34	0.81	−0.49	1.34	22.64	0.2	0.81	0.93	2.75%	20.00%
8	Treatment burden	7	235	0.35	0.95	−0.39	1.28	27.25	0.07	0.68	0.87	1.20%	14%
9	Prioritization	4	235	0.37	0.61	−0.41	1.27	12.94	0.37	0.61	0.75	0.00%	19.00%
10	Causal links	3	235	0.49	0.9	−0.48	1.26	14.68	0.1	0.49	0.8	0.00%	33.00%
11	Activity limitation	3	235	0.39	0.4	−0.63	1.4	7.13	0.62	0.72	0.74	0.00%	29.00%
12	Summary scale	22	235	0.02	1.88	−0.38	1.03	28.25	0.02	0.79	0.81	1.09%	2.60%
	**Ideal Values**			**0**	**<1.4**	**0**	**<1.4**		**>0.01**		**ns**	**<5%**	**<10%**

Key : SD = Standard Deviation; PSI = Person Separation Index; α = Cronbach's Alpha.

**Figure 1:**
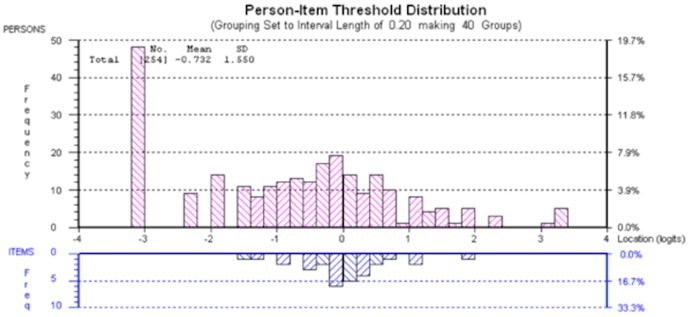
Person distribution for ‘Emotional Representations’ Scale. A large group of patients, represented by bars above the *x*-axis, fall between −1.6 and −3.2 logits, which were outside the measurable range of the scale, represented below the *x*-axis of the figure.

In spite of the scale's excellent model fit, a large proportion of the sample scored at the lower extreme of the scale (21.22%). Figure 1 shows a large group of patients falling between −1.6 and −3.2 logits, which were outside the measurable range of the scale (represented below the *x*-axis of the figure). Individual item fit statistics are displayed in Table 4.

**Table 4:** Fit of the MULTIPleS items to the Rasch model.

	Location	SE	FitResid.	ChiSq.	Prob.
Emotional Representations Scale					
Having more than one condition makes me more unhappy	−0.36	0.07	0.63	3.58	0.31
Having more than one condition makes me more anxious	−0.09	0.07	1.49	1.73	0.63
Having more than one condition makes me more frustrated	0.24	0.06	−0.27	3.37	0.34
Having more than one health condition makes me feel sad	−0.08	0.06	−0.96	4.65	0.2
Having more than one condition make me more irritable	0.01	0.06	−1.04	7.19	0.07
If I feel sad or depressed, managing all my conditions is a struggle	0.29	0.06	1.25	8.51	0.04
Treatment Burden Scale					
Taking different medications for each of my conditions has caused me problems	−0.4	0.1	2.04	4.55	0.21
Having more than one condition makes my treatments less effective	0.37	0.12	−0.88	7.55	0.06
It is difficult to take all my medications the way I am supposed to	0.32	0.11	−1.58	6.53	0.09
Having more than one condition makes it difficult to get the best available treatment	0.03	0.1	−0.76	1.75	0.63
I don't like mixing medications for different conditions	−0.36	0.11	1.15	0.64	0.89
I feel so overwhelmed by the treatment for one condition that it is hard to manage any others	0.04	0.11	1.62	1.78	0.62
Prioritisation Scale					
One of my conditions is more serious than the others	−0.17	0.09	1.28	2.64	0.45
One of my conditions has more of an impact on my life	−0.53	0.09	−0.28	7.58	0.06
One of my conditions dominates the others	0.59	0.08	−0.14	4.61	0.2
One of my conditions is more worrying than the others	0.1	0.08	0.19	6.8	0.08
Causal Links Scale					
The caused of my conditions are linked	−0.26	0.1	0.18	3.98	0.26
One of my conditions has caused another	0.2	0.1	0.55	3.56	0.31
My conditions interact with each other	0.06	0.1	1.29	1.76	0.62
Activity Limitation Scale					
Time spent managing my conditions has made it more difficult to carry out my usual activities	−0.03	0.1	0.36	2.55	0.47
Time spent managing my conditions has reduced my social life	0.09	0.1	0.6	4.38	0.22
Spending time managing my conditions has limited my activities	−0.06	0.1	0.23	1.97	0.58

#### Treatment burden scale

**Figure 2:**
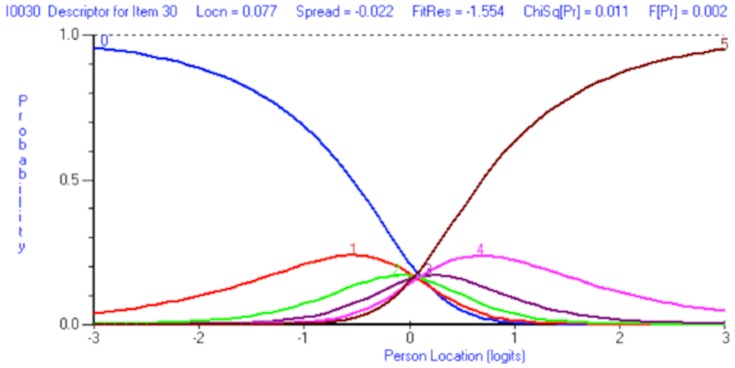
Category response thresholds for item 30. Disordered response thresholds are evident for item 30.

The eight-item ‘Treatment burden’ scale did not fit the Rasch model (χ^2^(21) = 166.35, p<0.0001). Unlike the Emotional Representations scale, category thresholds were disordered for every item within the scale, with a strong bias towards the first and last response category (‘Strongly agree’ and ‘Strongly disagree’) indicating that patients did not discriminate between six levels of agreement with each item statement. Figure 2 shows the disordered response categories for item 30 “Difficult to get the best treatment”. Thresholds 1 through 4 are disordered because at no point on the *x*-axis (person location) are they the most probable response option (*i.e.* they do not cross above the probability curves for response options 0 and 5).

**Figure 3:**
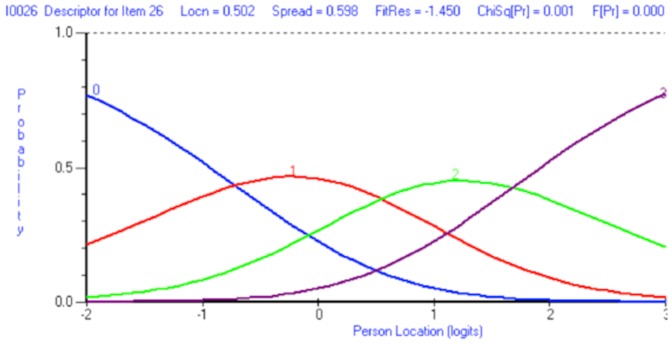
Category response options for item 26. Following rescoring, response thresholds are correctly ordered for item 26.

A revised scoring schedule was adopted for all the items within the scale, collapsing categories 1 and 2 together as well as 3 and 4, creating an effective 4-point Likert response structure scored 0-1-1-2-2-3. No items displayed disordered thresholds after being rescored, an example of correctly ordered thresholds for item 26 ‘Treatment less effective’ are given in Figure 3.

Rasch model fit was slightly improved by rescoring thresholds but high fit residuals for item 33 (‘Follow advice’, Fit Residual = 5.45) were still evident. By deleting item 33, model demands were met; including local independence of items, absence of DIF, ordered thresholds and acceptable reliability and unidimensionality (see Table 3, Analysis 2). A large proportion of patients scored at the lower extreme of the scale with almost of a third of all patients disagreeing with each item (32%). Individual item fit statistics are displayed in Table 4.

#### Prioritising conditions scale

The six-item ‘Prioritising conditions’ scale did not fit the Rasch model (χ^2^(18) = 76.34, p<0.0001). Misfit appeared to be driven by disordered thresholds for all items in the scale; with a strong bias toward the extreme response options (‘strongly disagree’ and ‘strongly agree’). The same rescoring protocol that was used for the ‘Treatment burden’ scale was adopted, leading to an effective 4-point Likert scale with correctly ordered thresholds. Item 17 displayed high fit residual following rescoring and was removed. Model fit was improved slightly but a high fit residual for item 13 was still apparent. Removal of item 13 led to good model fit including local independence of items, absence of DIF, ordered thresholds and good reliability and dimensionality (see Table 3, Analysis 3). Individual item fit statistics are displayed in Table 4.

#### Causal links scale

The three items of the Causal Links scale showed reasonable fit to the Rasch model (χ^2^(9) = 18.45, p = 0.03). Category thresholds for the scale were badly disordered for every item; the same rescoring protocol was adopted for this scale as had been used in the previous two analyses. Model fit was greatly improved and the demands of the Rasch model were satisfied (see Table 3, Analysis 4) dimensionality, absence of DIF and local dependency and acceptable reliability (see Table 3, Analysis 5). Individual item fit statistics are displayed in Table 4.

#### Activity limitations scale

The three item ‘Activity limitations’ scale displayed good fit to the Rasch model (χ^2^(9) = 13.73, p = 0.13). However category thresholds were disordered for all three items within the scale. Model fit was improved following rescoring in the same manner as the other scales including excellent dimensionality, absence of DIF and local dependency and acceptable reliability (see Table 3, Analysis 5). Individual item fit statistics are displayed in Table 4.

#### Summary Scale (Perceived importance of multimorbidity)

Following Rasch analysis of the five scales for the MULTIPleS measure, a higher-order summary scale was investigated to ascertain if the total score from all the items could produce meaningful measurement. Following subtesting, the summary scale showed excellent fit to the Rasch model (χ^2^(15) = 24.89, p = 0.05). When the scale was arranged in this manner scale targeting was excellent, with fewer than 2% of patients outside the measurable range of the scale. The scale was free of local dependency between subtests or DIF, had correctly ordered thresholds was reliable and unidimensional (Table 3, Analysis 6).

Analysis of mean item locations showed the relative positions of each of the scales when measured along the same linear continuum (see Figure 3). The ‘Prioritising conditions’ subscale represented the most readily affirmed scale (−0.50 logits) whilst the ‘Treatment Burden’ scale represented a greater degree of ‘perceived importance of multimorbidity’ (0.43 logits).

**Figure 4:**
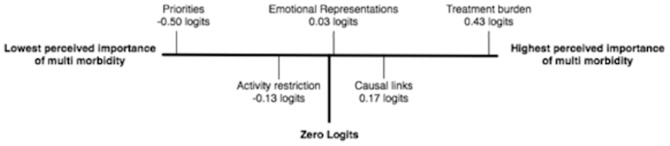
Illustration of logit (*i.e.* perceived importance of multimorbidity) value for each subtest in the Summary scale. When measured along the same linear continuum (perceived importance of multimorbidity) mean location of each subscale can be directly compared. By convention, in Rasch analysis the Summary Scale is centred around zero logits.

The final items that comprise the MULTIPleS measure, along with scoring information can be found in Additional Materials 1. Details of individual item fit statistics are given in Table 4.Figure 4 displays the relative location of the MULTIPleS subscale on the underlying ‘perceived importance of multimorbidity’ continuum that is measured by the Summary scale.

#### Validation Analysis

The data from the validation sample for each of the examined scales were fitted to the Rasch model. All scales fit the Rasch model at the 1% significance level and displayed similar reliability, unidimensionality and extreme scores as the evaluation sample (Table 3, Analyses 7–12).

**Figure 5:**
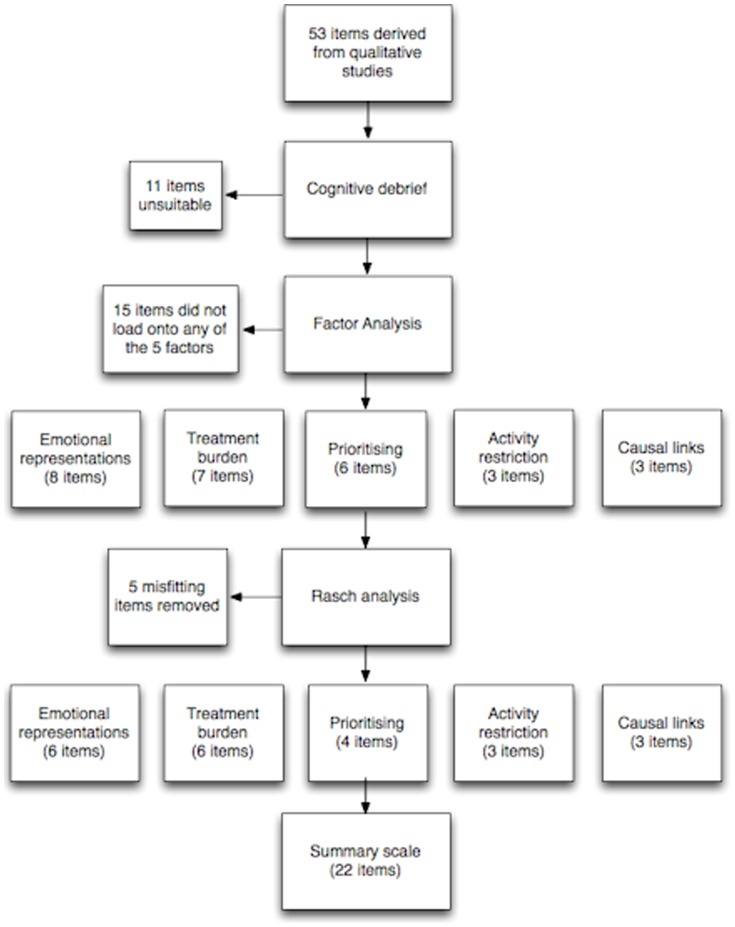
Analysis summary.

A summary of the stages of the analysis, detailing how many items were removed at each stage is given in Figure 5.

#### Test-Retest Analysis

Test-retest reliability was acceptable, with correlation coefficients above .50 for all scales (See Table 5.)

**Table 5:** Test-Retest correlations between MULTIPleS subscales.

Scale	Spearman's rho
Emotional Representations	0.69
Treatment Burden	0.63
Prioritsation	0.54
Causal Relationships	0.65
Activity Limitation	0.60
Summary Scale	0.70

#### External construct validity

Cross-sectional Spearman's Rho correlations between MULTIPleS, Brief Illness Perceptions Questionnaire (bIPQ), The Health Education Impact Questionnaire (heiQ) and the Hospital Anxiety and Depression Scale (HADS) are shown in Table 6. The strongest relationships are evident between MUTLIPleS and emotional domains from other measures.

**Table 6:** Construct validity of the MULTIPLES scale against external measures.

Construct	Causal links	Activity Restriction	Prioritising	Treatment Burden	Emotional Representations	Total Score
Brief Illness Perception Questionnaire (bIPQ)						
Impact of illness	0.28	0.45	0.42	0.32	0.51	0.5
Timeline of illness			0.12			
Perceived control of illness	−0.05	−0.19	−0.17	−0.16	−0.23	−0.21
Efficacy of treatment	−0.02	−0.17	−0.12	−0.16	−0.17	−0.17
Experience of symptoms	0.18	0.38	0.36	0.25	0.42	0.41
Concern	0.29	0.35	0.4	0.28	0.52	0.48
Understanding of illness				−0.11		
Emotional affect	0.37	0.46	0.44	0.44	0.7	0.62
Total score	0.29	0.37	0.4	0.26	0.47	0.45
Health Education Impact Questionnaire (heiQ)						
Emotional	0.48	0.61	0.53	0.57	0.81	0.77
Constructive attitude shift	−0.29	−0.47	−0.32	−0.45	−0.58	−0.54
Skills and knowledge acquisition	−0.18	−0.27	−0.24	−0.37	−0.48	−0.4
Self monitoring and insight	−0.1	−0.08	−0.07	−0.27	−0.27	−0.2
Hosptial Anxiety and Depression Scale (HADS)						
Anxiety	0.37	0.52	0.41	0.49	0.72	0.65
Depression	0.35	0.53	0.43	0.5	0.69	0.64
Psychological Distress	0.38	0.55	0.43	0.52	0.74	0.67

All values significant at p<0.05, non-significant values suppressed.

#### Raw score to interval score conversion

Table 7 displays a nomogram by which ordinal scores gained from the MULTIPle scales can be transformed to interval level data, provided there are no missing data and that data are normally distributed. Researchers may use this nomogram to change raw scores obtained from completed questionnaires to interval scores suitable for parametric statistics.

**Table 7:** Nomogram for calculating interval level score for MULTIPleS.

Raw Score		Scale					Raw Score	Scale
	Summary Scale	Emotional	Treatment burden	Prioritization	Causal links	Activity limitation		Summary Scale
0	0	0	0	0	0	0	36	36.83
1	6.77	3.68	2	1.3	1.5	1.27	37	37.17
2	11.22	6.06	3.44	2.29	2.62	2.24	38	37.51
3	14.13	7.59	4.46	3.04	3.45	2.98	39	37.84
4	16.32	8.72	5.29	3.69	4.16	3.67	40	38.18
5	18.1	9.62	5.99	4.29	4.84	4.37	41	38.52
6	19.58	10.36	6.62	4.88	5.56	5.19	42	38.85
7	20.87	11.01	7.2	5.49	6.39	6.19	43	39.19
8	22.02	11.58	7.75	6.16	7.51	7.48	44	39.53
9	23.05	12.09	8.27	6.94	9	9	45	39.87
10	23.99	12.57	8.79	7.97			46	40.22
11	24.84	13.02	9.31	9.55			47	40.57
12	25.64	13.44	9.85	12			48	40.93
13	26.39	13.85	10.43				49	41.3
14	27.08	14.24	11.06				50	41.67
15	27.74	14.64	11.81				51	42.06
16	28.36	15.03	12.73				52	42.45
17	28.94	15.42	14.06				53	42.86
18	29.5	15.82	18				54	43.27
19	30.03	16.24					55	43.7
20	30.54	16.68					56	44.15
21	31.03	17.14					57	44.62
22	31.5	17.64					58	45.11
23	31.95	18.19					59	45.62
24	32.38	18.83					60	46.16
25	32.8	19.56					61	46.72
26	33.21	20.47					62	47.31
27	33.6	21.63					63	47.94
28	34	23.26					64	48.61
29	34.37	25.86					65	49.32
30	34.74	30					66	50.09
31	35.11						67	50.92
32	35.46						68	51.82
33	35.81						69	52.81
34	36.16						70	53.9
35	36.5						71	55.13
							72	56.52
							73	58.15
							74	60.09
							75	62.51
							76	65.75
							77	70.63
							78	78

## Discussion

### Summary

The Multimorbidity Illness Perceptions Scales (MULTIPleS) were developed to measure patient illness perceptions in the presence of multimorbidity. By application of the Rasch model, we have demonstrated that the constituent MULTIPle scales are reliable, unidimensional and fit the Rasch model. The presence of a higher-order scale, which consisted of all the items from the five disparate scales, was confirmed using the Rasch model.

### Limitations of the study

Our response rate in a postal questionnaire among patients with long-term conditions living in the community replicates our previous surveys of this type in the UK [20], [26], [44]. However, it does leave open the possibility of response bias, which means that the present analysis may not generalise to all patients with multimorbidity. The use of a postal survey will be least accessible to those with low levels of education or health literacy, which may be important given the socioeconomic patterning of multimorbidity [9]. We were not ethically permitted to access medical records for non-responders so were unable to test for systematic biases between responders and non-responders. Response rates of this level are conventional for questionnaire research carried out in primary care [26], [45], [46].

We chose patients with a range of conditions to maximise the relevance of the results, but little is known about how illness perceptions vary among different conditions and different combinations, and the current results will need to be replicated with other conditions to assess wider relevance.

One limitation of the Rasch model, in its current form, is the use of the Chi-Square fit statistic to assess model fit. The Chi-Square statistic is somewhat limited, and users of other modelling techniques, such as Structural Equation Modelling tend to prefer other indicators of model fit, such at the Root Mean Square Error of Approximation (RMSEA) [47]. The Chi-square statistic is sensitive to both sample size [48] and data distribution [49].

### Interpretation of the results

The results of the factor analysis were broadly in line with the initial conceptual grouping of items. The original conceptual grouping of items around ‘Consequences’ was split empirically into factors relating to ‘Emotional Consequences’ and ‘ Activity limitations’. The dimensions of ‘Priorities’ and ‘Causal links’ were confirmed, although our original groupings around ‘Coherence’ and ‘Synergies’ were combined in the factor ‘Treatment Burden’ in the empirical analyses.

Response categories were disordered for many of the items that were analysed using Rasch analysis. Many of the items with disordered categories appeared to show a strong bias towards responses at the extremes of the scale. This may be an artefact of the large percentage of items that were scored 0 or may represent a genuine dichotomisation of these issues for patients living with multimorbidity (i.e. they are affected or they are not) without lesser degrees. Current work is planned to investigate the effect of different Likert-response formats on the MULTIPle scales [50].

Whilst fit to the Rasch model was achieved for all the scales, for each scale there was a group of patients who fell at the floor of the scale, suggesting that these patients did not form illness perceptions related to those specific domains (*e.g.* did not think their illnesses were casually related). Analysis of person-item threshold locations revealed that whilst each of the scales did have a group of patients that scored at the extreme of the scale, the scale was generally well targeted for the remainder of patients. No floor effect was present when all of the scale were added together to make a summary scale; indicating that whilst the formation of illness perceptions in multimorbidity is heterogeneous our summary scale was able to accurately measure the level of illness perceptions formed by all but a minority (2%) of respondents. The variability in the perceptions of multimorbidity is supported by research findings from our previous qualitative study [20]. Further work could be conducted to investigate the possibility of adding new items that may capture illness perceptions for those patients who create very weak perceptions of multimorbidity.

The MULTIPleS scales showed positive relationships between the Brief Illness Perception Questionnaire, the Health Education Impact Questionnaire and the Hospital Anxiety and Depression Scale. This analysis revealed that MUTLIPleS appears to be correctly profiling the emotional impact of living with multiple long-term conditions.

The presence of a sizeable floor effect for the individual scales did affect the scale reliability when measured using person separation index (Cronbach's alpha is unaffected by the presence of a floor effect). Further work will assess characteristics of patients with low scores on the scales to assess whether they display particular clinical, demographic or psychological characteristics and explore reasons for such responses among patients.

Investigation of a potential higher-order factor revealed that the individual domains of the MULTIPle measure could be added together to form a summary scale with good measurement properties that we labelled ‘Perceived importance of multimorbidity’. The summary scale had excellent psychometric properties. Using the super-ordinate scale it was possible to display the five subscales along a linear continuum. Ranking items in this order showed that respondents were most likely to form representations about prioritising one condition. The most ‘difficult’ representation to form was that of burden caused by multimorbidity, indicating that it is patients who have formed a number of illness perceptions who are likely to consider that multimorbidity causes additional burden. This finding may guide further work that explores the development of illness perceptions in patients with multimorbidity. It is an important empirical question as to whether the subscales or the higher-order scale predict health behaviour and outcomes more effectively

Test-retest reliability was assessed using a random subsample of patients who originally completed the scale at baseline. Test-retest reliability was acceptable, though correlation coefficients were somewhat below those reported in previous Rasch analyses [41] indicating some variability in multimorbidity illness perceptions over time. Test-restest was conducted over a 4-week period and natural variation in illness perceptions over this period may artificially reduce test-retest validity s cores. However, results from the test-retest analysis were congruent with previous research developing questionnaires to measure illness perceptions in single conditions [17].

We suggest that MULTIPleS Summary scale will be suitable for most clinical and research purposes. It provides a reliable unidimensional measure of illness perceptions that is capable of providing accurate comparisons between patients and within patients over time. We suggest that the MULTIPleS subscales primarily useful for profiling patient illness perceptions, allowing the researcher or clinician to see identify which perceptions about multimorbidity are salient to the patient or patients they are interested in.

Given the proportion of extreme scores on some subscales (e.g. treatment burden and causal links), we suggest that the MULTIPleS subscales are best used at this time to provide descriptive data on patient illness perceptions in these areas, prior to further research (such as further patient interviews) on the meaning and validity of these extreme scores and the profiles of patients who respond in this way.

### Conclusions

In summary the MULTIPleS questionnaire is a series of five unidimensional measures of illness perceptions in multimorbidity that can be added to create a total score representative of a higher-order factor of multimorbidity illness perceptions.

Future analyses will further assess the construct validity of the scales by assessing their relationships with external measures, including demographic, clinical and psychological variables. The critical test then relates to the validity of the scale in predicting outcomes such as self-management, health related quality of life and health care utilisation in patients with multimorbidity, which might provide the basis for the future development and evaluation of interventions to reduce the impact of multimorbidity.

## Supporting Information

File S1 **Items from the MULTIPleS scale with scoring information.**
(PDF)
