# Supplementary material for: Development of a Multimorbidity Illness Perceptions Scale (MULTIPleS)
Source: PLoS One. 2013 Dec 20;8(12):e81852. doi: 10.1371/journal.pone.0081852 (PMC3869652; doi:10.1371/journal.pone.0081852)
Supplement: File S1 — Items from the MULTIPleS scale with scoring information. (PDF) [file pone.0081852.s001.pdf]

The Multimorbidity Illness Perceptions Scale (MULTIPleS)

These questions are about the thoughts and feelings that people with multiple long-term conditions sometimes experience. Please indicate how much you agree with each statement by circling one number.

		Strongly Disagree →			Strongly Agree
1	One of my conditions is more serious than the others.	0	1	2	3
2	Time spent managing my conditions has made it more difficult to carry out my usual activities.	0	1	2	3
3	I feel so overwhelmed by the treatment for one condition it is hard to manage any others.	0	1	2	3
4	The causes of my conditions are linked.	0	1	2	3
5	It is difficult to take all my medications the way I am supposed to.	0	1	2	3
6	Time spent managing my condition has limited my activities.	0	1	2	3
7	One of my conditions is more worrying than the others.	0	1	2	3
8	Taking different medications for each of my conditions has caused me problems.	0	1	2	3
9	I don't like mixing medications for different conditions.	0	1	2	3
10	Having more than one condition makes my treatments less effective.	0	1	2	3
11	One of my conditions has caused another.	0	1	2	3
12	One of my conditions dominates the others.	0	1	2	3
13	My conditions interact with each other.	0	1	2	3
14	Having more than one condition makes it difficult to get the best available treatment.	0	1	2	3
15	Time spent managing my conditions has reduced my social life.	0	1	2	3
16	One of my conditions has more of an impact on my life.	0	1	2	3

		Strongly Disagree →					Strongly Agree
17	Having more than one condition makes me unhappy.	0	1	2	3	4	5
18	Having more than one condition makes me more anxious.	0	1	2	3	4	5
19	Having more than one condition makes me angry or frustrated.	0	1	2	3	4	5
20	Having more than one health problem makes me feel sad.	0	1	2	3	4	5
21	Having more than one condition makes me more irritable.	0	1	2	3	4	5
22	If I feel sad or depressed, managing my conditions is a struggle.	0	1	2	3	4	5

Notes on scoring the MULTIPleS scale.

The MULTIPleS scale is divided into five discrete domains of Emotional representations, Treatment burden, Prioritisation, Causal Relationships and Activity restriction. The items that consist of these scales are given below

Treatment burden = Items 3, 5, 8, 9, 10, 14

Prioritisation = Items 1, 7, 12, 16

Causal Relationships = Items 4, 11, 13

Activity Restriction = Items 2, 6, 15

Emotional representations = Items 17, 18, 19, 20, 21, 22

Summary scale (importance of multimorbidity)= Items 1-22

SPSS Macros for computing domain scores

If item variables are entered as MULT1-MULT22, the following macros will calculate domain scores for the MULTIPleS scales

Treatment Burden

COMPUTE TBURD=MULT3+MULT5+MULT8+MULT9+MULT10+MULT14

Prioritisation

COMPUTE PRIOR= MULT1+MULT7+MULT12+MULT16

Causal Relationships

COMPUTE CAUSR= MULT4+ MULT11+ MULT13

Activity Restriction

COMPUTE AREST=MULT2+MULT6+MULT15

Emotional Representations

COMPUTE EMOREP= MULT17+MULT18+MULT19+MULT20+MULT21+MULT22

Summary Scale

COMPUTE MULTSUM=TBURD+PRIOR+CAUSR+AREST+EMOREP
